# The lived experiences of newly qualified midwives in supporting women during labor and birth: A hermeneutic phenomenological study in Malta

**DOI:** 10.18332/ejm/200073

**Published:** 2025-02-05

**Authors:** Jeanette Gauci, Rita Pace Parascandalo

**Affiliations:** 1 Mater Dei Hospital, Ministry of Health, Msida, Malta; 2 Department of Midwifery, Faculty of Health Sciences, University of Malta, Msida, Malta

**Keywords:** newly qualified midwives, junior midwives, early career midwives, lived experiences, labor and birth care, hermeneutic phenomenological study

## Abstract

**INTRODUCTION:**

The well-being of midwives impacts the quality of care provided during labor and birth, influencing maternal and neonatal outcomes. It is crucial that newly qualified midwives (NQMs) feel confident in managing labor and childbirth, to foster positive experiences for both mother and child. This study aims to explore and understand the lived experiences of NQMs caring for women during labor and birth.

**METHODS:**

A Heideggerian hermeneutic phenomenological approach was used to explore the experiences of ten NQMs within two years post-qualification, working in the Central Delivery Suite (CDS) of Malta's main public hospital. Purposive sampling identified participants, and data were collected through one-time, semi-structured, in-depth interviews conducted in private settings with the first author (JG) between December 2021 and January 2022. The interviews followed a self-designed schedule in English (JG, RPP) and were audio-recorded. Reflective journaling was maintained throughout. Analysis was guided by van Manen’s six-step method, with hermeneutic philosophy and Willian Bridges' transition model informing the interpretation.

**RESULTS:**

Two main themes emerged: 1) ‘Baptism of fire’ and 2) ‘Containing the fire’. ‘Baptism of fire’ encapsulates the initial challenges NQMs faced, including feelings of being overwhelmed and unprepared. ‘Containing the fire’ highlights how NQMs developed strategies to adapt, growing more confident and effective in their roles. These experiences were shaped by their training exposure to CDS and the level of support from colleagues during their transition.

**CONCLUSIONS:**

The findings highlight the need for supportive environments, extended CDS placements and structured guidance through preceptorship. Enhancing NQMs' transition to practice has implications for midwifery education, policy, and practice.

## INTRODUCTION

Childbirth is a pivotal and emotional event in a woman’s life, influencing her self-perception and relationships with her partner and family^1^. Research indicates that a midwife’s well-being directly affects the quality of care provided to women in labor and newborn outcomes^2^. Therefore, it is essential for newly qualified midwives (NQMs) to feel confident in caring for women during labor and birth, to ensure a positive childbirth experience. NQMs are crucial to the progression of midwifery^3^. Their ability to transition effectively from student to registered midwife essentially impacts the profession and maternity health services^4^. A smooth transition is vital for job retention, particularly as many countries face midwifery shortages^5^. Existing literature describes this transition as a ‘reality shock’ due to increased responsibilities^4,6,7^. Therefore, this period must focus on fostering NQMs’ confidence and competence^8^.

In Malta, the undergraduate midwifery program at the University of Malta (UOM) equips students to meet the competencies outlined by the International Confederation of Midwives^9^ and comply with European Union directives, including Midwives Directive 80/154/EEC Article 4, Directive 2005/36/EC Article 42 (as amended by Directive 2013/55/EU), and the Health Care Professional Act [cap.464, p4, art IV, 23(5)]. The program combines theoretical instruction, clinical teaching, and placements, primarily conducted at Malta’s main public hospital.

This study explores the lived experiences of NQMs caring for women during labor and birth. The birthing unit is widely regarded as the most challenging maternity setting for NQMs. Building on existing literature, this study delves into the meaning of NQMs’ feelings and experiences as they assist women during labor and birth, moving beyond the traditional focus on the transition from student to employed midwife. It highlights how NQMs manage both normal physiological labor and birth and obstetric emergencies in Malta, aiming to address their needs within this demanding environment.

## METHODS

### Reflexivity

Reflexivity is particularly essential in hermeneutic research, as the researcher engages with the phenomenon through own preunderstandings^10^. In hermeneutic phenomenology, the researcher becomes an integral part of the research tool^10^.

To address this, the primary researcher (JG) maintained a personal journal throughout the study. This practice allowed for iterative reflection, tracking the study’s progression, and ensuring that interpretations were grounded in the participants’ experiences rather than assumptions. The journal served as a reminder to focus on genuinely understanding the participants and heightened the researcher’s awareness of potential preconceptions during the research process. The primary researcher (JG) conducted the interviews, while both researchers (JG, RPP) contributed to the study’s concept and design, data collection, analysis, and interpretation.

### Study design

A qualitative hermeneutic phenomenological approach was adopted, drawing on Heidegger’s interpretive philosophy to explore the ‘meaning of being’^11^. Hermeneutics involves various philosophical concepts, including being, being-in-the-world, encounters with entities, spatiality, temporality, and care structure^12^. Key Heideggerian notions such as Dasein, thrownness, attunement to mood, solicitude, and temporality^13^ were utilized to interpret NQMs’ lived experiences. Additionally, Bridges’ transition model, which describes a three-phase process of adapting to change: Ending/losing/letting go, The neutral zone, and The new beginning, was used to guide the interpretation of findings^14,15^.

### Setting

The study was conducted at the Central Delivery Suite (CDS) in Malta’s main public hospital, after the necessary permissions and ethics approval were granted. All participants had completed a six-month rotation within their first two years post-registration at the CDS. Face-to-face interviews were conducted with each participant by one of the researchers (JG) and held in a private room within the hospital between December 2021 and January 2022.

### Participants and sampling

The sampling method was aligned with the chosen research approach^16^. In hermeneutic research, the concept of Dasein, the lived existence of participants, is crucial, as the goal is to explore and uncover the meaning of an experience. Consequently, participants who had direct experience of the phenomenon being studied were recruited to address the research question ^10,17,18^.

In this study, no age restrictions were applied to the recruitment of NQMs. However, all participants were required to have completed a rotation placement in the Central Delivery Suite (CDS) within their first two years post-registration. Midwives qualified for more than two years were excluded to ensure participants could vividly recall and articulate their lived experiences.

Using purposive sampling, ten participants were recruited. All individuals who consented to participate remained engaged throughout the study, each contributing through a semi-structured interview.

### Data collection

Data were collected through one-time, face-to-face interviews. The interview schedule was self-designed in the English language by both researchers. The questions were crafted to align with the study’s aims and objectives. A reflective journal was maintained throughout the research process to support reflexivity and ensure the integrity of data interpretation.

Interviews, lasting 45 minutes to an hour, facilitated the capture of both verbal and non-verbal communication^19,20^. All interviews were transcribed verbatim, with non-verbal cues incorporated to enhance the depth of interpretation. Data analysis was conducted iteratively, starting with each individual interview, and continuing through transcription, coding, and thematic development^10,21^.

### Data analysis

Transcripts were manually analyzed using van Manen’s^22^ principles of thematic analysis, focusing on both explicit and implicit themes to uncover the core meanings of NQMs’ experiences. This method was selected as it aligns closely with the principles of hermeneutic phenomenology, where the researcher is considered an integral tool in the study^10^.

Data analysis commenced before the formal data collection process, with the primary researcher (JG) documenting thoughts and preunderstandings in a reflexive diary^10^. In hermeneutic phenomenology, analysis is a reflective and iterative process, referred to as the hermeneutic circle^10^. Themes and patterns emerged inductively from the data through repeated engagement with the transcripts, involving multiple readings to deepen understanding.

The analysis procedure was thoroughly documented, with an acknowledgment of potential researcher biases. To ensure confirmability, verbatim quotes were included to demonstrate that the interpreted findings accurately reflected participants’ experiences. Confirmability was further strengthened by maintaining a reflexive journal throughout the study, ensuring transparency and rigor in the research process^10^. The themes and subthemes are given in Tables 1 and 2.

**Table 1:** Data analysis for the first theme ‘Baptism of fire’

*Newly qualified midwives’ views*	*Keywords*	*Concepts*	*Subthemes*
*‘It is a very busy environment when compared to the wards for example there is not much of a routine. At Central Delivery Suite you never know if you are going to start the day at 7, or later on. Because, ehm, you might have a mother coming at the beginning of your shift, or she comes in later. So, the routine is very different. Sometimes, you might even not have time to have a break not like maybe in other wards. So ehm even mentally it is quite exhausting as well.’* (Participant 1)	Busy ward	Demanding ward environment	Challenging environment
	The place with the highest expectations		
	Bad connotations about ward	Mentally/physically exhausting	
	Unpredictable ward events		
	Learning while working		
*‘… we do our deliveries in our third year and we have a whole two months practicing our deliveries. In our fourth year, we didn’t have any placements at the Central Delivery Suite so we didn’t have any deliveries. I mean we practiced skills because we were practicing for our finals, but we weren’t practicing as many skills as if we were doing the deliveries. Then I had another year to go to Central Delivery Suite so I was quite a bit in shock that I would, ehm, forget some skills or for example when performing a vaginal examination, that I wouldn’t do it right and obviously if another person examines (the woman) after me that it would be a whole, ehm, different findings…’* (Participant 4)	Lost touch	Feelings of incompetence	Feelings of a junior midwife
	Lost practice		
	Feel alone	Feeling stranded	
	Feel lost		
	Feel overwhelmed		
	Fear of the unknown	Always on edge	
	Fear of failing		
	Putting oneself down /not worthy of profession		
	Whole responsibility on your shoulder	Realities of becoming a midwife	
	Not reaching expectations		
	Difficult to adapt to change		
	Giving best care to the mother		
*‘We try to avoid maybe speaking about sometimes other junior midwives. At times I’ve heard things about junior midwives who are my friends. And I say “if she found out, if she hears what they are saying about her” and then thinking at the same time I wonder what they say about me (uncomfortable giggle) and the same time you feel, ehm, that type of fear. And it does affect you mentally and how you work with mothers because if you don’t feel comfortable with the staff, you fear that they might say something about you. Because without wanting to, you doubt yourself and you don’t give your 100 percent to the mothers you are caring for. Because you’re constantly with that doubt “Am I doing this right? Or am I not? Are they going to say something if I do this and not that?” That constant feeling you know what I mean? That if they talk something about you.’* (Participant 1)	Being the outsider	Team inclusion	Trying to fit in
	Going with the current		
	Working against the current		
	Caught in the middle		
	Choosing your people		
	Belittled	Superior and dominating personalities	
	Feel let down		
	Working under pressure		
	Victim of gossip		
	Facing traumatic experiences and not understood		
	Staff don’t remember they were in the same boat		

**Table 2:** Data analysis for the second theme ‘Containing the fire’

*Newly qualified midwives’ views*	*Keywords*	*Concepts*	*Subthemes*
‘You’ll do a lot of skills in a short period of time for example, I don’t know during labor you do a lot of skills so VEs, catheters if you need. In other wards, for example, VEs you don’t do a lot of them, maybe in antenatal mothers when you need to. But at labor ward you do them a lot even multiple times during the day. So, I think if you learn that skill it will stay with you. And even if you need to do a VE in another ward, if you worked at CDS I think it would help to do them more comfortable in other wards, rather than just not doing them at all and then you have to do them in other wards.’ (Participant 10)	It is the heart of midwifery	True meaning of midwifery	Of the essence
	Individualized one-to-one care		
	Steppingstone to grow as a midwife	Learning and growing environment	
	Missed/gained opportunities		
‘The very first delivery I was so focused on making sure that I’m doing everything right, when you are not that confident that building the relationship with the mother, unfortunately, wasn’t my top priority. At times doing a delivery, you feel very connected and emotional. In the very first delivery I didn’t feel that because I was so focused on the birth … So, I think that the emotional and social aspect (of birth) was a bit low on that side so I wouldn’t say that it was one of the best. But then after 3 months in my rotation, I felt more confident. I also had some of these complications where I felt that I am capable of handling things independently more. Then I started getting more confident with the work (not only look at the mechanism of birth but notice that dealing with a woman) and even the emotional aspect of bonding with the mother increased.’ (Participant 8)	Applying knowledge and practice	Things falling into place	Better with time
	Learning by doing		
	Taking a stand/believing in self	Growing as a midwife	
	Relationship with mothers		
	Feel accomplished and proud of self		
‘There needs to be this more supportive environment for us NQMs. And we have to keep in mind that everyone progresses at different paces as well, because it doesn’t mean that every midwife get used to the delivery suite in 3 months, that everyone is going to adapt like that. Everyone is different and everyone learns and gets used to things differently, there are some who take longer to get there.’ (Participant 1)	Guidance	Supportive measures	An eased transition
	Reassurance		
	Given feedback		
	Given space to reflect/vent		
	Support system		
	Gain trust and respect	Colleagues’ support	
	Allowed to fly and not be grounded		
	Not compared to others		
	Welcomed		
	Comfort from peers		
	More time in ward	Environment support	
	Simulations		
	Ward orientation		
	Structured plan		
	Weaned into it		

### Ethical considerations

Ethical approval for the study was gained on 17 June 2021 (V_15062020 8848). The participants were approached by an intermediary person, who provided them with the information letter, with a clear explanation of the study’s purpose and details. Prior to each interview, participants signed a consent form to confirm their willingness to participate. Although they were informed in advance about the audio recording, explicit permission was sought again before starting the recordings.

In compliance with the General Data Protection Regulation (GDPR) and national legislation, participants retained the right to access, rectify, and, where applicable, request the erasure of data concerning them. These measures ensured the study adhered to ethical and legal standards for participant rights and data protection.

## RESULTS

### Demographic characteristics

All participants in the study were of female gender aged 23–26 years. They had completed a six-month placement in the Central Delivery Suite (CDS) as part of their two-year rotation period. One participant was assigned to the CDS as her first placement immediately upon starting her employment. The remaining participants (n=9) completed their CDS placements either midway through their rotation (n=7) or in the final phase (n=2) after being assigned to other wards first. During the interview the majority of the participants (n=7) had completed their rotation while 3 of the participants were in the middle of their rotation.

### Themes

The analysis of the narratives from the NQMs and the first author’s (JG) preunderstandings revealed two overarching themes, each encompassing three subthemes. Despite the uniqueness of each midwife’s experience in the CDS, shared elements emerged that were shaped by their personal characteristics, previous work experiences, interactions with colleagues, the women they cared for and the evolving nature of their professional identity. The two themes identified were ‘Baptism of fire’ and ‘Containing the fire’. The themes and subthemes are found in Tables 1 and 2.

*Baptism of fire*

The theme ‘Baptism of fire’ encapsulates the experiences of NQMs as they begin their placements in the Central Delivery Suite (CDS). Here, ‘Baptism’ symbolizes the initiation into their new professional roles, while ‘fire’ represents the various challenges and hurdles they face. These obstacles range from navigating a hectic work environment and managing intense emotions to the difficulty of integrating into a new team. The NQMs viewed completing their CDS placement as crucial to shaping them into the midwives they aspired to be, despite the trials they encountered. This theme emerged from three subthemes: 1) Challenging environment, 2) Feelings of a junior midwife, and 3) Trying to fit in.

Participants consistently identified the CDS as the most demanding ward within the maternity setting. One participant described the unpredictability and intensity of the ward, noting that:

*‘… it keeps you a bit on edge, a bit tense, not knowing what to expect, because as you know CDS it’s all about the unexpected so you don’t know what you are going to face when you go in … you can have ambulance calls, you can have patients who come in who are about literally about to push the baby out. You can have patients who unfortunately have their baby died in utero so, it’s not always like a pretty picture every day … and even if you have the smoothest labor sometimes problems can arise during the second stage, even during the third stage sometimes.’* (Participant 5)

While NQMs entered the Central Delivery Suite (CDS) confident in their knowledge, they often felt they lacked the experience and practical expertise possessed by their more senior colleagues. They grappled with the transition from the protected role of a student to the fully accountable position of a qualified midwife, responsible for the care of both mother and newborn during labor and birth. This sudden shift brought about fears of failing to meet expectations and being perceived as incompetent or untrustworthy. Another participant highlighted the urgency of learning on the job, noting:

*‘… there are times that the first time you need to perform a skill is in an emergency …’* (Participant 3)

The intense emotional and mental demands of the CDS often left NQMs feeling drained, which impacted their interactions with family members at home. A participant reflected on this exhaustion:

*‘I ended up going home crying that I’ve done something wrong (looks very sad) … I also asked my relatives what they thought, for me it was the time that I was exhausted the most when going home, and they (her family) always said that it was during the rotation at CDS that I always went exhausted at home.’* (Participant 4)

NQMs described a relentless cycle of replaying work scenarios in their minds, questioning their actions, and reflecting on any criticism received, which led to increased stress and dread for their next shift. A participant expressed this continuous mental strain:

*‘I think it’s because of remembering the feelings … of going back from work thinking about everything. “Did I do everything?”, “Did I do everything right?”, “Did I cater to all the mothers’ needs?”, “Did I forget something?” (as she says it her voice takes on a higher pitch and her words are faster as if showing how she used to feel) … So, my work-life balance wasn’t that good (laughs) and it was very stressful (in a lowered voice) … it was that feeling of going back home and still not being able to put work aside. I don’t think it’s good to live like that (laughs nervously).’* (Participant 7)

At the outset of their placement, many NQMs felt lost and overwhelmed, particularly when they lacked the necessary support from colleagues. A participant captured this sense of vulnerability:

*‘Most of the midwives take care of junior midwives very well because they support you, they help you out, they teach you. But then you find other midwives that sort of, ehm, do not bother basically and leave you up to it. And I think that is the most stressful part of it. That having a senior midwife with you or an in-charge leaving you alone. It’s like moving around without support. Having no legs and moving around without support, I usually feel like this when I am not supported.’* (Participant 6)

The pressure to perform competently, even under stress, added to their anxiety. A participant reflected on the need for reassurance from experienced colleagues, stating:

*‘Although I did have another rotator who I could speak to, and we could share our experiences with each other. Sometimes it’s good to have, you know, maybe a senior midwife or someone who has been working there a long time, to tell you that you’re doing well. It makes you feel a bit better. I think there needs to be those words of encouragement as well that I felt were very lacking … there should be those positive affirmations … I think though it is because of the environment (CDS), it’s a bit more stressful when we want to get everything right because the area is more intense. But there needs to be more support.’* (Participant 1)

Many NQMs felt the need to mask their insecurities and maintain a facade of confidence, especially during interactions with doctors or when giving reports during ward rounds. A participant described the anxiety of these moments, saying:

*‘I went home lots of times, and I cried, or I went in the treatment room, and I cried, especially if maybe a doctor or even midwives shouted at me or if I felt like overwhelmed. Like I still remember this, but even giving over on that desk waiting for someone to shout at me (shows great sadness). That is how I used to feel with certain head of shifts. I used to say “Oh my god it’s that shift, I have to give over. Let me check that everything is perfect because I know that I am going to hear a complaint or something, in front of everyone”.’* (Participant 9)

A profound concern for NQMs was the fear of not being accepted by their colleagues. They often felt like outsiders, needing to prove themselves repeatedly to gain acceptance. They felt torn between striving to be the midwife they aspired to be and conforming to the expectations of their colleagues. A participant highlighted the pressure to conform, saying:

*‘… There’s a lot of pressure from colleagues to transfer the mother to the postnatal ward as quickly as possible. I feel that this is unfair, you know? You need to give that time for the mother and the father to get used to the baby. That first hour after birth is very important (makes emphasis on this point). However, I feel there were a lot of pressures to transfer the mother very quickly, even sometimes when we weren’t busy … Sometimes you feel like you even need to lie to the mother because you need to transfer her to the other ward because of pressures from other staff. Not because necessarily, they would need the room.’* (Participant 1)

Overall, the theme ‘Baptism of fire’ underscores the intense and often overwhelming experiences of NQMs as they transition into their roles within the CDS. These experiences, marked by both professional challenges and personal introspection, highlight the need for better support systems to ease this crucial transition period for new midwives.

*Containing the fire*

The theme ‘Containing the fire’ represents the stage in the NQMs’ journey when they begin to manage and control the ‘fire’ – the difficulties, obstacles, and fears – highlighted in the previous theme. As NQMs gain experience and confidence, they start to suppress the overwhelming challenges that once hindered their transition from being a junior to a more confident midwife. They come to realize that having successfully completed their pre-registration course and received their certification, they possess the necessary knowledge and competencies to perform effectively as midwives. Participants also recognize the CDS as a crucial ward in their development into the midwives they aspire to become, with things beginning to align more clearly in their professional practice. With appropriate support, the ‘fire’ can indeed be contained, easing their transition. This theme is derived from three subthemes: 1) Of the essence, 2) Better with time, and 3) An eased transition.

For NQMs, the CDS was seen as embodying the essence of midwifery, providing a critical foundation for their growth in the role. They viewed it as a steppingstone, packed with unique experiences that could only be encountered within this particular placement. The CDS allowed them to continuously practice essential skills. A participant reflected:

*‘I think overall I left Central Delivery Suite feeling more confident as a midwife and I could see myself growing. I think that was the main place where I felt myself grow as a midwife. You feel as I am learning more here, it’s not that in other places [wards] you don’t learn but it is one of the main places (CDS) where you feel like I am doing the job of a midwife and I am learning more. That was mainly it.’* (Participant 9)

NQMs realized that the experiences gained at the CDS ultimately enhanced their capabilities in other maternity wards, preparing them for various situations that deviated from the norm. A participant shared this sentiment, stating:

*‘Sometimes when I look back I say, “you know, if I went through that I will manage to go through everything (laughs)”.’* (Participant 3)

As they spent more time in their roles, NQMs noticed that their experiences and knowledge began to integrate more seamlessly into their daily practice. They grew more comfortable with both the staff and the ward setting, gaining practical experience that allowed them to combine theoretical knowledge with practical skills. A participant described her early experience:

*‘The first two or three deliveries that I encountered I was more on edge. Because, I had to be quick with the things that I need to do.’* (Participant 1)

However, all participants agreed that being welcomed and oriented by colleagues on their first day would have greatly facilitated their adaptation, helping them familiarize themselves with the equipment, instruments, and local ward guidelines. A participant emphasized this need, saying:

*‘Having someone welcome you (laughs) and even explain where the stuff is. Going in the nursery and telling you “Listen here is the stuff that you need, this is the cupboard (laughs) that you need to open and get the stuff from’ (laughs)”, you know? Because everywhere is different. You’d know where everything is in most delivery rooms, but I think, for example, the nursery you’d have no idea. Even having a welcome (laughs) will help.’* (Participant 3)

Participants expressed a desire not to be compared with other NQMs, noting that individuals adapt at different paces and have varying reactions to situations. They appreciated having peers to share experiences with, finding comfort in mutual understanding, which contrasted with some senior midwives who seemed to lack empathy, perhaps having forgotten their own early experiences. A participant captured this feeling, saying:

*‘… but I felt like there was everyone at the desk and I am here like having this major issue, like come on! I mean and these are people who are not only my colleagues but my friends, “come help me!” you know? “You don’t remember the days when you were like me?” (laughs as if to hide the betrayal she felt at that moment).’* (Participant 9)

All participants wished they had been assigned a preceptor or a senior midwife at the start of their placement to gradually help them become more independent. They felt the need for a safe space to reflect on their experiences and vent their feelings, especially after traumatic events such as an intrauterine death (IUD) or a cord prolapse, where they often felt unsupported and misunderstood by colleagues. A participant suggested a need for more understanding and reflective practice, stating:

*‘I think other staff need to be more understanding, that you’re coming to something so new, that you haven’t done deliveries in a long time, ehm, so definitely one needs support … most of the time I had to ask for reassurance … So I think the fact that maybe the seniors ask you “Listen are you okay? How are you doing?”. It makes a big difference and maybe even towards the end of the rotation they (managers/head of shift) ask you “Is there anything we can improve on?”. Because I think the problem with our department is that we get stuck and we don’t ask and reflect, so yes, I think that is something we could do.’* (Participant 9)

While NQMs needed guidance, they also wanted the opportunity to work autonomously, particularly when tasks carried no significant risks to the mother or infant. They felt that being trusted to make decisions would help them grow more confident and feel more respected. A participant articulated this balance between supervision and autonomy, saying:

*‘So maybe at first, okay, they shadow you and they teach you, but then towards the end if you’re doing well … they shouldn’t keep influencing their way of working on yours … towards the end of the rotation you expect that you are respected a bit more, in a way like your own autonomy. You are an autonomous midwife and that’s your way …’* (Participant 8)

Participants felt constrained by the six-month duration of their CDS placement. They believed this was insufficient time to reach their full potential as midwives within such a complex and demanding environment. As they began to feel more confident and capable, they were required to move to a different setting, leaving them feeling unsatisfied and longing for more time to develop further. A participant captured this sentiment, stating:

*‘I would say that I needed a bit more time, I think six-months it’s not enough, you need at least nine months if not a year. Because the first three months it’s running in, that’s what I felt it was. So by the end, you know, you start feeling to be more confident and you really start enjoying the work, way after three months in my personal experience.’* (Participant 8)

Overall, ‘Containing the fire’ reflects the NQMs’ journey toward managing the intense challenges of their new roles, gaining confidence, and growing into their positions as midwives. With adequate support and time, they began to see themselves not as novices overwhelmed by the ‘fire’ but as capable professionals capable of ‘containing’ it.

## DISCUSSION

Based on the main findings of this study, this discussion is structured under three subheadings: 1) The birthing unit, 2) The midwives’ journey, and 3) Needs to succeed. The interpretation of findings is guided by Heidegger’s phenomenology and Bridges’ transition theoretical framework. Heidegger’s phenomenology focuses on understanding the ‘meaning of being’ through interpretation^11^. Key philosophical concepts include being, being-in-the-world, being-with, encounters with entities, spatiality, temporality, and the care structure^12^. From these, five hermeneutic notions: Dasein, thrownness, attunement to mood, solicitude, and time, were selected to interpret the lived experiences of being NQMs, providing deeper insights into their journey.

Additionally, Bridges’ transition model framework complemented this interpretation. Developed to help individuals and organizations navigate change^14^, this model outlines a three-phase process: 1) Ending/losing/letting go, 2) The neutral zone, and 3) The new beginning^15^. These phases offer a lens to understand how NQMs adapt to their new roles and responsibilities.

### The birthing unit

Participants perceived the CDS (Central Delivery Suite) as the most intimidating and challenging maternity setting. This aligns with findings from Clements et al.^4^, Avis et al.^23^, Fenwick et al.^24^, and Sheehy et al.^25^, who also reported that NQMs found birthing units particularly daunting during their transition from students to qualified professionals. Participants described the CDS as a steep learning curve, where they were required to rapidly acquire and apply new skills, some of which were unfamiliar. Kool et al.^26^ and Sheehy et al.^25^ similarly identified the simultaneous learning of multiple skills as a significant source of stress during the transition from student midwives to registered midwives. Interestingly, literature indicates that even participants who had prior nursing experience before becoming registered midwives found maternity wards challenging and intimidating^27-29^.

In the context of hermeneutic phenomenology, with regard to Heidegger’s concept of ‘thrownness’^12,13^, Thomson^30^ suggests that individuals are ‘thrown’ into a world with pre-existing values and norms, influencing their behaviors and experiences. Applied to NQMs, this notion reflects their experience when starting their midwifery careers, particularly in the CDS, a setting with its own culture and established practices. The concept of ‘thrownness’ is evident in their continuous exposure to new and challenging situations, requiring them to provide care for mothers with diverse needs while adapting to the CDS’s unique environment. This notion of ‘thrownness’ can also be integrated with Bridges’ transition model. According to Bridges^31^, change is situational and often beyond an individual’s control. The first phase of Bridges’ framework, ‘Ending/losing/letting go’, aligns with ‘thrownness’, as NQMs were placed in the CDS per hospital protocol, regardless of their apprehension. They were compelled to relinquish their previous roles as student midwives, moving away from the familiarity of other maternity wards and taking on full responsibility for the care of their patients.

As time progressed, participants began to adapt to the CDS, experiencing a sense of fulfilment as they provided more woman-centered care during labor and birth. This adaptation corresponds with the third phase of Bridges’ transition model, ‘The new beginning’, which involves embracing new values, understandings, and a sense of purpose^31^. Over time, NQMs’ perceptions of the CDS shifted, and they viewed it as the most satisfying ward, offering the best opportunity to build trusting relationships and provide holistic care for mothers.

However, these findings contrast with those of Griffiths et al.^27^, who reported heightened concerns among NQMs working in hospital-based maternity settings regarding continuity of care. The study of Griffiths et al.^27^ highlighted that those participants with prior experience in caseload midwifery during their student years felt unable to provide consistent care in a hospital environment. In contrast, participants in the present study, who had only experienced a local hospital setting, did not express the same concerns.

Furthermore, the final phase of Bridges’ transition model, ‘The new beginning’, became evident as participants neared the end of their six-month placement, recognizing the significance of working at the CDS as a pivotal step toward becoming proficient midwives. The CDS provided NQMs with essential learning opportunities that contributed to their professional growth, enabling them to become more competent in various maternity settings. Some participants noted that, despite working in other ward settings, they felt they truly became midwives after their CDS placement. This is a unique finding of this study, possibly because previous research primarily focused on the transition from student midwives to NQMs, rather than on the actual experiences of NQMs caring for women during labor and birth.

### The midwives’ journey

Findings highlight the experiences of NQMs as they transition from being students to practicing midwives, specifically focusing on the challenges they face during their placement in the Central Delivery Suite (CDS). This journey is marked by fear, anxiety, and an essential adjustment period, influenced by both past and present experiences.

NQMs’ fear and anxiety are not only shaped by their current roles but are also deeply influenced by their experiences as student midwives in the CDS. Those who had negative experiences as students carried this apprehension into their new roles, fearing a repeat of those challenges. This anticipation of a negative experience at the CDS was unique to this study, as existing literature did not explore the impact of past student experiences on NQMs. However, research by Cazzini et al.^32^, Saliba^33^, and Sheehy et al.^25^ found that familiarity with the work environment as students, helped midwives adjust better when they returned as professionals. This aligns with Heidegger’s^13^ notion of time, where past experiences influence the present and future, suggesting that time is not linear but a continuous interplay of past, present, and future experiences.

The study found that experiences at the CDS could critically impact NQMs’ career choices. Negative experiences could deter them from returning to the CDS, which could have broader career implications given the importance of the CDS in the Maltese public hospital system. The Clements et al.^4^ study showed that some midwives chose to work in different maternity settings with continuous care models after negative experiences in hospital birthing units. This suggests a potential career shift for NQMs to avoid environments that trigger negative emotions or fear.

NQMs expressed fear of causing harm to mothers and babies, making incorrect decisions, and being unable to handle obstetric emergencies. This fear of the unknown, as described by Lovecraft^34^, is a powerful emotion that profoundly influences NQMs’ experiences. Dahlen and Caplice^35^ also found that midwives, regardless of experience level, feared making mistakes that could harm the mother or baby, highlighting that this fear persists throughout a midwife’s career.

The emotional burden of these fears did not remain confined to the workplace but extended into NQMs’ personal lives, negatively impacting their well-being and daily activities. This finding is supported by Kitson-Reynolds et al.^36^, Kool et al.^26^, and Norris^37^, who also noted that work-related stress could lead to personal life disruptions, affecting sleep and overall health. This aligns with Heidegger’s^13^ concept of attunement to mood, where an individual’s mood is shaped by their experiences and the world they inhabit. The NQMs’ experiences reflect a mood of fear and anxiety, which impacts their ability to function effectively in their roles.

The study also highlighted feelings of isolation and being overwhelmed among NQMs, particularly when they did not receive adequate support from colleagues. NQMs found the transition from student to professional midwife difficult, often feeling unprepared for the responsibilities they were now expected to handle. This mirrors findings by Sheehy et al.^25^ and Lennox et al.^38^, who reported that NQMs often felt unsupported and overwhelmed, which hindered their ability to perform effectively. The concept of ‘Reality shock’ described by Wain^6^ captures this phenomenon, where the harsh realities of the workplace create a noteworthy adjustment challenge for newly qualified professionals.

NQMs expressed notable insecurities regarding decision-making, particularly in skills such as vaginal examinations (VEs) and cardiotocography (CTG). They feared making mistakes that could lead to adverse outcomes, reflecting the critical and subjective nature of these skills. This uncertainty is characteristic of the ‘Neutral zone’ in Bridges’ transition model^31^, where individuals are caught between their old and new roles, struggling to adapt to their new responsibilities. Similarly, Hobbs^39^ and Kool et al.^26^ found that NQMs struggled to build confidence in their midwifery skills, particularly those requiring subjective judgment.

The study revealed that NQMs felt that their past experiences as students, where they were often overshadowed by senior midwives and not given opportunities to make decisions, negatively impacted their confidence and preparedness. This lack of experience left them feeling unprepared for their new roles, aligning with Heidegger’s^13^ notion of time, where past experiences shape present and future actions. The philosophical notion of solicitude^13^ further illustrates how lack of supportive preceptorship (‘leaping in’) can leave NQMs feeling dependent and lacking confidence, while supportive guidance (‘leaping ahead’) can empower them.

NQMs reported feeling constrained by senior midwives who were resistant to change and often took over situations, which prevented them from practicing autonomously and applying evidence-based practices. This again reflects Heidegger’s notion of solicitude, where ‘leaping in’ undermines the confidence and autonomy of NQMs. In contrast, supportive colleagues who explained their decisions helped NQMs improve their decision-making skills and confidence, a finding which is congruent with those in Lennox et al.^38^ and Young et al.^40^.

The study also highlighted the hierarchical dynamics within the CDS, where NQMs often felt belittled and humiliated by more senior staff, impacting their willingness to ask for help or assert themselves. This corresponds with findings by Sheehy et al.^25^, Lennox et al.^38^, and Keeling et al.^41^, who noted that hierarchical structures and bullying behaviors can impact NQMs’ confidence and well-being, potentially leading to long-term physical and psychological effects.

Despite these challenges, NQMs gradually moved beyond the initial transitional phase, gaining a clearer understanding of their roles and responsibilities. This represents the final phase of Bridges’ transition model, ‘The new beginning’, where individuals begin to feel a sense of purpose and establish a new identity^31^. The study showed that with time and support, NQMs could overcome their fears and anxieties, develop confidence in their abilities, and become effective midwives, highlighting the importance of supportive guidance and a positive work environment.

### Needs to succeed

Newly qualified midwives (NQMs) indicated the essential factors required to feel confident, secure, and successful in their roles, especially during their initial placements in a clinical delivery suite (CDS). The study highlights the importance of structured support systems, including proper orientation, mentorship, supportive workplace relationships, and opportunities for reflection and debriefing. These elements contribute to a smoother transition and professional growth for NQMs. The findings align with existing literature, underscoring the importance of these supportive measures in enhancing NQMs’ experiences and reducing burnout^25,38^.

NQMs expressed a strong need for comprehensive orientation to the ward environment, even if they had previously experienced a placement there as student midwives. This re-orientation is crucial to alleviate anxiety and reduce feelings of being lost. Proper orientation involves familiarization with the ward setting, equipment, protocols, and guidelines, which helps NQMs feel secure and confident. This finding is consistent with studies by Avis et al.^23^, Lennox et al.^38^, and Sheehy et al.^25^, which also emphasize the importance of a well-structured induction process.

All participants in the study highlighted the importance of being assigned a preceptor or senior colleague when they started working in the CDS. Having someone to guide them helps ease their transition during the ‘Neutral zone’ phase of Bridges’ transition model^31^, providing a reliable source for advice in challenging situations. Preceptorship provides stability and reduces confusion caused by conflicting opinions among colleagues^6^. It also promotes professional development through role modeling, as described by Heidegger’s^13^ notion of ‘leaping ahead’. This need for preceptorship is supported by other studies, including Avis et al.^23^, Kool et al.^26^, Saliba^33^, and Wain^6^ who found that this enhances NQMs’ confidence and job satisfaction.

Building good relationships with colleagues is crucial for NQMs’ confidence and sense of security. Negative experiences, such as mistreatment by colleagues, can result in a negative placement experience. A supportive and welcoming workplace helps NQMs adapt more quickly, as noted in studies by Cazzini et al.^32^, Norris^37^, and Sheehy et al.^25^. These studies affirm that a positive, collaborative work environment is essential for a smooth transition and effective learning.

Participants emphasized the need for regular opportunities for debriefing and reflection to process challenging or stressful experiences. The lack of these opportunities can lead to burnout, stress, and frustration. This finding aligns with Doherty and O’Brien^42^, who identified a lack of debriefing and reflection as a prominent factor contributing to burnout among midwives, particularly those in high-pressure birthing units. Providing space for reflection not only alleviates stress but also supports learning and professional development, as supported by Lennox et al.^38^ and Sheehy et al.^25^.

While participants felt more comfortable and confident by the end of their six-month placement, they acknowledged that the duration was too short to fully consolidate their knowledge and skills. Longer placements would provide more opportunities to experience diverse situations and improve competence in providing woman-centered care, reflecting the transition to the ‘New beginning’ phase of Bridges’ transition model. Wain^6^ similarly found that NQMs needed more time in clinical settings to build confidence and gain comprehensive experience. Conversely, Avis et al.^23^ and Kool et al.^26^ reported that shorter rotations could lead to insecurity and hinder confidence-building.

### Strengths and limitations

In hermeneutic phenomenology, preunderstanding of the phenomenon is essential. The researchers, being midwives and former NQMs themselves, brought valuable insights into the participants’ lived experiences. This background enabled the researchers to act as integral tools in the study, enhancing the depth of interpretation. Additionally, detailed descriptions of the research process, study context, and participants’ characteristics, strengthen the study’s potential transferability. The study’s strengths lie in its qualitative approach, which facilitated the collection of rich, in-depth data from a small sample of participants. While the findings are not intended to be generalizable, they are transferable to settings with similar characteristics and organizational structures, particularly as the study is grounded in experiences from a single public hospital.

The interpretation of findings was conducted solely by the authors, and it is acknowledged that alternative interpretations might arise with different researchers. Additionally, the study could be potentially limited by selection bias, as most participants who agreed to participate reported negative experiences in their placements at the CDS. This may have influenced the findings, as those with positive experiences might have been less inclined to take part. Furthermore, recall bias poses a potential limitation, as participants’ memories of past experiences could be influenced by the passage of time or emotional intensity.

### Recommendations

To address the challenges faced by NQMs and to facilitate a smoother transition into their professional roles, the study offers several recommendations for educational and clinical placement settings. These include comprehensive orientation to the ward, structured induction programs, the involvement of a dedicated practice midwife and preceptors, clinical area simulations, extended rotation periods, and accessible emotional and psychological support from colleagues and other professionals. Additionally, regular feedback meetings and opportunities for constructive criticism are recommended to foster a more supportive and nurturing environment for NQMs. Implementing the ability of NQMs to transition effectively into their roles, ultimately helping them become the best midwives they can be in a healthier and more supportive setting.

## CONCLUSIONS

This study highlights the challenges that newly qualified midwives (NQMs) face in adapting to the intense environment and high stress levels of the Central Delivery Suite (CDS) during their placement rotations. The emotional toll of their experiences often affected their personal lives, compounded by unsatisfactory mentorship and inadequate support from colleagues both during their student placements and later as NQMs. Despite the difficulties encountered, participants recognized the CDS as the most fulfilling aspect of their work, viewing it as the core of midwifery that ultimately contributed to their growth and development as midwives.

## Data Availability

The data supporting this research cannot be made available for privacy or other reasons.
